# Secular trends in sickness absence among Swedish patients with ankylosing spondylitis and psoriatic arthritis

**DOI:** 10.1007/s00296-017-3809-z

**Published:** 2017-09-06

**Authors:** Christel Nielsen, Ingemar F. Petersson, Lennart T. H. Jacobsson, Anna Jöud

**Affiliations:** 1 Division of Occupational and Environmental Medicine, Department of Laboratory Medicine, Lund University, P.O. Box 188, 221 00 Lund, Sweden; 2 R&D Centre Skåne, Skåne University Hospital Lund, Lund, Sweden; 3 Epidemiology and Register Centre South, Skåne University Hospital Lund, Lund, Sweden; 4 Division of Orthopaedics, Department of Clinical Sciences Lund, Lund University, Lund, Sweden; 5 Department of Rheumatology and Inflammation Research, Sahlgrenska Academy, University of Gothenburg, Gothenburg, Sweden

**Keywords:** Ankylosing spondylitis, Psoriatic arthritis, Sickness absence, Secular trends, Epidemiology

## Abstract

The aim was to investigate whether secular trends in sickness absence (SA) were present in patients with ankylosing spondylitis (AS) and psoriatic arthritis (PsA) receiving their diagnosis between 2002 and 2011. A repeated cross-sectional study design was used. Patients were identified in the Skåne Healthcare Register (SHR). A washout period of 18 months was applied. The general population seeking health care was used as a reference cohort. SA data from 2003 to 2012 were obtained from the Swedish Social Insurance Agency and converted into net days of SA per year. Within diagnosis and sex, the average number of net days of SA during the calendar year following diagnosis was calculated and plotted against calendar year together with the corresponding SA of the age-standardized reference population. Linear regression on aggregated data, within diagnosis and sex, was applied to formally investigate differences in secular trends among patients and referents. There were 3173 patients and 992,502 referents. Among men diagnosed with AS, the average amount of SA declined by 8.1 net days per year in patients as compared with 2.4 in the referents (*p* = 0.01). Among PsA patients, the average amount of SA declined by 11.7 net days per year in women as compared with 2.7 in the referents (*p* < 0.001) and by 7.6 net days per year in men as compared with 1.9 in the referents (*p* < 0.001). Secular trends of declining SA were present among AS and PsA patients. Trends were also present among the referents, although not at all of the same magnitude.

## Introduction

Spondyloarthritis and its comorbidities are associated with impaired physical function [1], and reduced quality of life [2]. Besides the comorbidities typically related to spondyloarthritis, studies have also reported increased risks of cardiovascular disease and associated risk factors in ankylosing spondylitis (AS) and psoriatic arthritis (PsA) patients [3–5], and depression in AS patients [6]. Taken together, the consequences of AS and PsA impact on several aspects of affected individuals’ lives and restrict patients’ ability to participate in society, resulting in reduced work productivity and increased sickness absence (SA) [7, 8].

AS and PsA debut relatively early in adulthood and are often chronic of nature. Thus, the diseases have the potential to impair patients’ work ability throughout a large part of their working life, imposing financial consequences for the individual patient as well as society. Indeed, the cost of AS patients in rheumatologic care in southern Sweden has been shown to be three times higher than that of reference subjects from the general population, and the most important cost item is that related to sickness absence [9].

During the last decade, however, several structural changes have taken place that may affect the amount of SA experienced by AS and PsA patients. There has been extensive societal focus on SA. Swedish rules and regulations have been subject to revision and authorities have launched initiatives to reduce the SA burden. The Swedish Social Insurance Agency administers economic compensation in connection with disease or injury for individuals of working age that legally live or work in Sweden. The Swedish Social Insurance Agency has become more stringent in its assessment of the sickness absence note as a result of the policy changes [10], and it is now required that diagnosis, functional impairment, and activity limitation must be logically linked for SA to be approved [11]. New effective pharmacological treatment regimens, i.e., tumour necrosis factor (TNF)-alpha inhibitors and other biological pharmaceuticals, were introduced. TNF-alpha inhibitors have been associated with achieving partial remission in patients diagnosed with AS [12], and with positive effects on musculoskeletal and cutaneous outcomes, function, quality of life, fatigue, and radiographic progression in patients diagnosed with PsA [13]. Indeed, better patient functioning following treatment with TNF-alpha inhibitors is supported by the previous reports of reduced SA in both AS [14, 15] and PsA patients [16, 17].

The objective of the study was to explore the presence of secular trends in SA between 2003 and 2012 among newly diagnosed AS and PsA patients in southern Sweden and in the general population seeking health care. A second objective was to assess whether such secular trends among patients were related to changes in the proportion of patients experiencing SA, in the amount of SA experienced by patients with some amount of SA, or both. We hypothesised to find a declining secular trend among AS and PsA patients and that the trend would be more pronounced than that observed in the general population seeking health care.

## Materials and methods

### Data sources

#### Definition of patients and referents

A repeated cross-sectional study design was used. Patients were identified in the Skåne Healthcare Register (SHR), which contains all in- and out-patient visits to public and private health-care providers for 1.3 million individuals residing in southern Sweden, by searching for ICD-10 codes related to AS and PsA (i.e., M45, L40.5, and M07.0-3). To be valid for inclusion, the diagnosis was required to be set by a rheumatologist between 2002 and 2011. The year of diagnosis defined a patient’s index year. The ICD-10 code received at this occasion was considered a patient’s diagnosis. To identify patients with newly diagnosed disease, and thus avoid carry-over effects on SA from a more generous policy during the 1980s, a washout period of 18 months was applied during which the patients were not allowed to have had an AS or PsA diagnosis.

Further inclusion criteria were that patients were required to have been resident in the Skåne region during the washout period, the index year, and the calendar year following the index year; patients should be 17–66 years of age during the index year; and patients should be alive at the end of the calendar year following the index year. Yearly data on residency by December 31st were available from the Swedish Population Register, a demographic database covering information on all inhabitants’ vital events managed by Statistics Sweden.

The general population in Skåne was considered the reference cohort. Individuals with at least one visit to a clinic during the calendar year and with a diagnosis set by a physician were identified on a yearly basis in the Skåne Healthcare Register (SHR). The rationale was to avoid bias caused by differences between referents and patients with respect to their likelihood to seek health care. Further inclusion criteria were that referents should be 18–67 years of age; resident in the Skåne region; and alive by December 31st according to the Swedish Population Register. The reference cohort was intended to reflect the general population in the Skåne region and thus contained also the AS and PsA cases.

#### Sickness absence

Information on SA between 2003 and 2012 was obtained from the Swedish Social Insurance Agency. Sickness compensation is granted by the Swedish Social Insurance Agency in case of work incapacity lasting 14–364 days within a 15-month period. Disability pension can be granted by the Swedish Social Insurance Agency in case of long-lasting work incapacity. Both types of benefits are based on the amount of reduction of daily work capacity and may be granted for 25, 50, 75, or 100% of full working time. Furthermore, the two may be combined on the same day, although never exceeding 100%. Hereafter, SA will be taken to include days compensated with either sickness compensation, disability pension, or both. The first day of SA is not compensated and days 2–14 days are reimbursed by the employer. SA periods with shorter duration than 14 days were thus not included in the study.

Net days of SA per year, i.e., expressed as full-time SA, were calculated by multiplying crude days of SA by its degree and then summing them over calendar year. Our outcome of interest was net days of SA per calendar year. As such, it was necessary to have information on SA for full years and we, therefore, focused on SA during the calendar year following the index year.

### Statistical analyses

The proportions of women and men diagnosed with AS and PsA, respectively, with some amount of SA during the calendar year following the index year were compared using the Pearson *χ* ^2^ test. Thereafter, the data were stratified on diagnosis and sex. Within strata, differences in age at diagnosis in patients with and without SA during the calendar year following diagnosis were assessed using the non-parametric Mann–Whitney *U* test.

Within each diagnosis and sex stratum, the average number of net days of SA during the calendar year following the index year, together with approximate confidence intervals, were calculated and plotted. The average number of net days of SA per calendar year in the female and male reference cohorts, respectively, was age-standardized according to the female and male AS and PsA cohorts. Four age strata were used: ≤39, 40–49, 50–59, and ≥60 years.

Linear regression on aggregated data, within diagnosis and sex, was applied to formalize the exploration of (1) the overall secular trends in net days of SA during the calendar year following the index year and to (2) verify if the overall secular trends could be explained by changes in the proportion of patients that experienced some amount of SA during the calendar year following the index year and/or (3) changes in the amount of SA experienced by patients with some amount of SA during the calendar year following the index year. The following models were used:

1. Net days of SA = cohort (patient or reference) + calendar year (2003–2012, continuous variable) + cohort × calendar year.
2. Proportion = calendar year (2003–2012, continuous variable).
3. Net days of SA = calendar year (2003–2012, continuous variable).

Results were obtained using the SAS software, version 9.4 (SAS Institute Inc., Cary, NC, USA). *P* values below 0.05 were considered statistically significant.

## Results

### Descriptive statistics

A total of 3173 patients were identified and the reference cohort included 992,502 individuals during the study period. Irrespective of diagnosis, a larger proportion of women than men experienced SA during the calendar year following the index year. During the calendar year following the index year, 54% of women, irrespective of diagnosis, experienced some amount of SA. The corresponding figures in men were 36% in AS and 37% in PsA (Table 1). Patients with some amount of SA were older when they received their diagnosis as compared with patients without SA. This was particularly pronounced in those diagnosed with AS.

**Table 1:** Proportions of 3173 patients in southern Sweden with ankylosing spondylitis (AS) and psoriatic arthritis (PsA) with sickness absence (SA) during the calendar year following diagnosis and their age at diagnosis

	With SA		Without SA	
	*n* (%)	Age at diagnosis, mean ± SD	*n* (%)	Age at diagnosis, mean ± SD
AS				
Women	167 (54***^a^)	46 ± 11***^b^	142 (46)	40 ± 13
Men	228 (36)	49 ± 11***	409 (64)	42 ± 12
PsA				
Women	659 (54***)	50 ± 10***	560 (46)	47 ± 13
Men	368 (37)	50 ± 10***	640 (63)	47 ± 12

***p < 0.001

^a^Within-diagnosis comparison of the proportion of female and male patients with SA

^b^Within diagnosis and sex comparison of the age at diagnosis in patients with and without SA

**Fig. 1:**
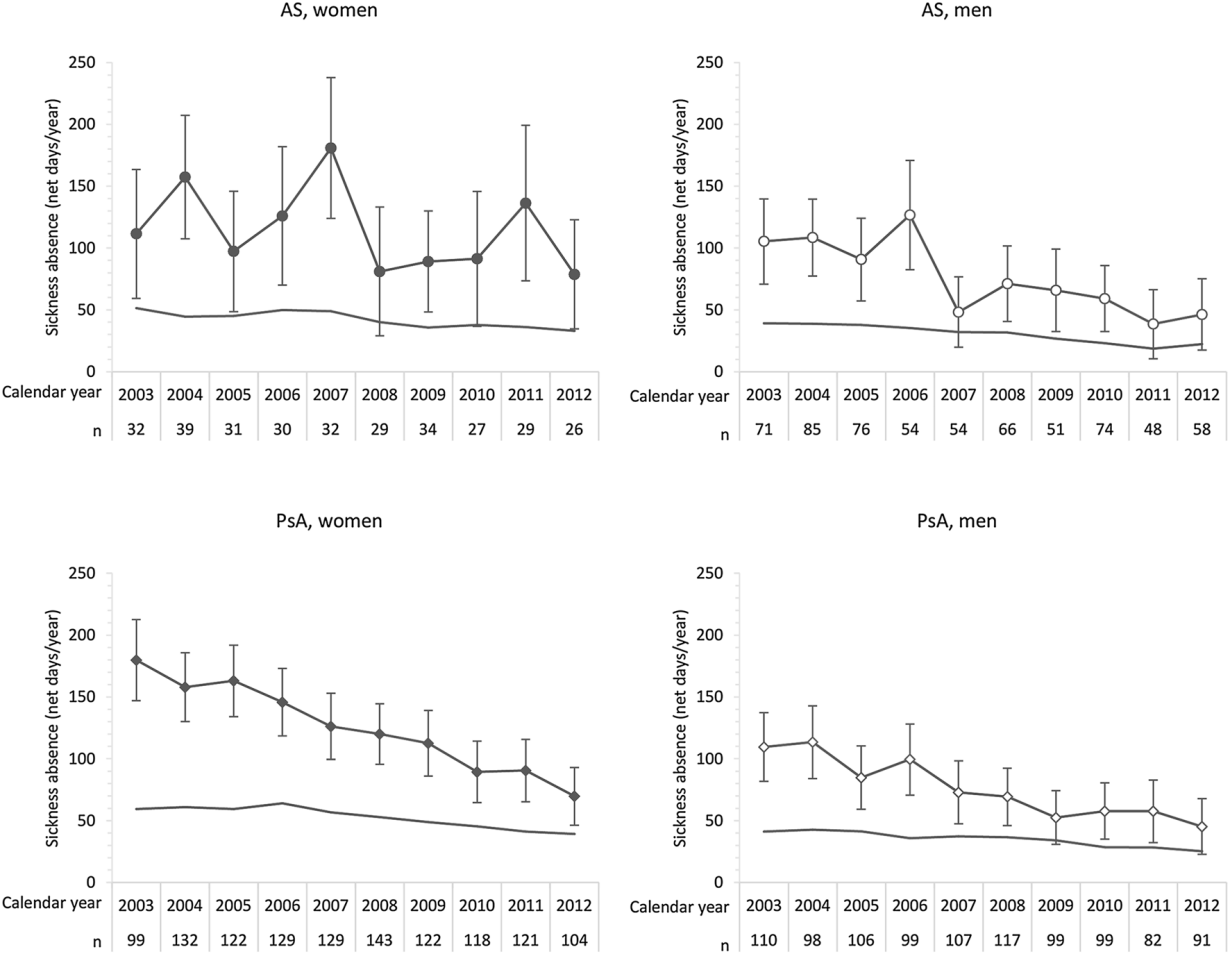
Sickness absence (average number of net days per year) during the calendar year following diagnosis in women and men with ankylosing spondylitis (AS) and psoriatic arthritis (PsA) receiving their diagnosis between 2002 and 2011, together with 95% confidence intervals. The number of patients diagnosed each year is given below the *x*-axis. The solid curves represent the corresponding figures of the, within diagnosis and sex, age-standardized reference population seeking health care

The results support the presence of a declining secular trend in the average number of net days of SA among men diagnosed with AS and among all patients diagnosed with PsA (Fig. 1; Table 2), whereas the trend in the reference population was less pronounced. In absolute figures, the average number of net days of SA decreased from 111 to 79 in women and from 105 to 46 in men diagnosed with AS. In PsA patients, the corresponding changes were from 180 to 70 in women and from 110 to 45 in men between 2003 and 2012. In 2012, the average number of net days of SA experienced by women diagnosed with AS and PsA during the calendar year following the index year was above 70, whereas the corresponding figures for men were below 50.

**Table 2:** Results of the linear regression models estimating the yearly change in the average number of net days of sickness absence during the calendar year following diagnosis in patients diagnosed with ankylosing spondylitis (AS) and psoriatic arthritis (PsA), compared with the reference population

Change of net days of SA in patients with SA and referents (model 1)			
	*β*-coefficient		*p* value^a^
	Patients	Referents	
AS, women	−4.1	−1.9	0.56
AS, men	−8.1	−2.4	0.01
PsA, women	−11.7	−2.7	<0.001
PsA, men	−7.6	−1.9	<0.001

^a^ *p* value of interaction term

**Fig. 2:**
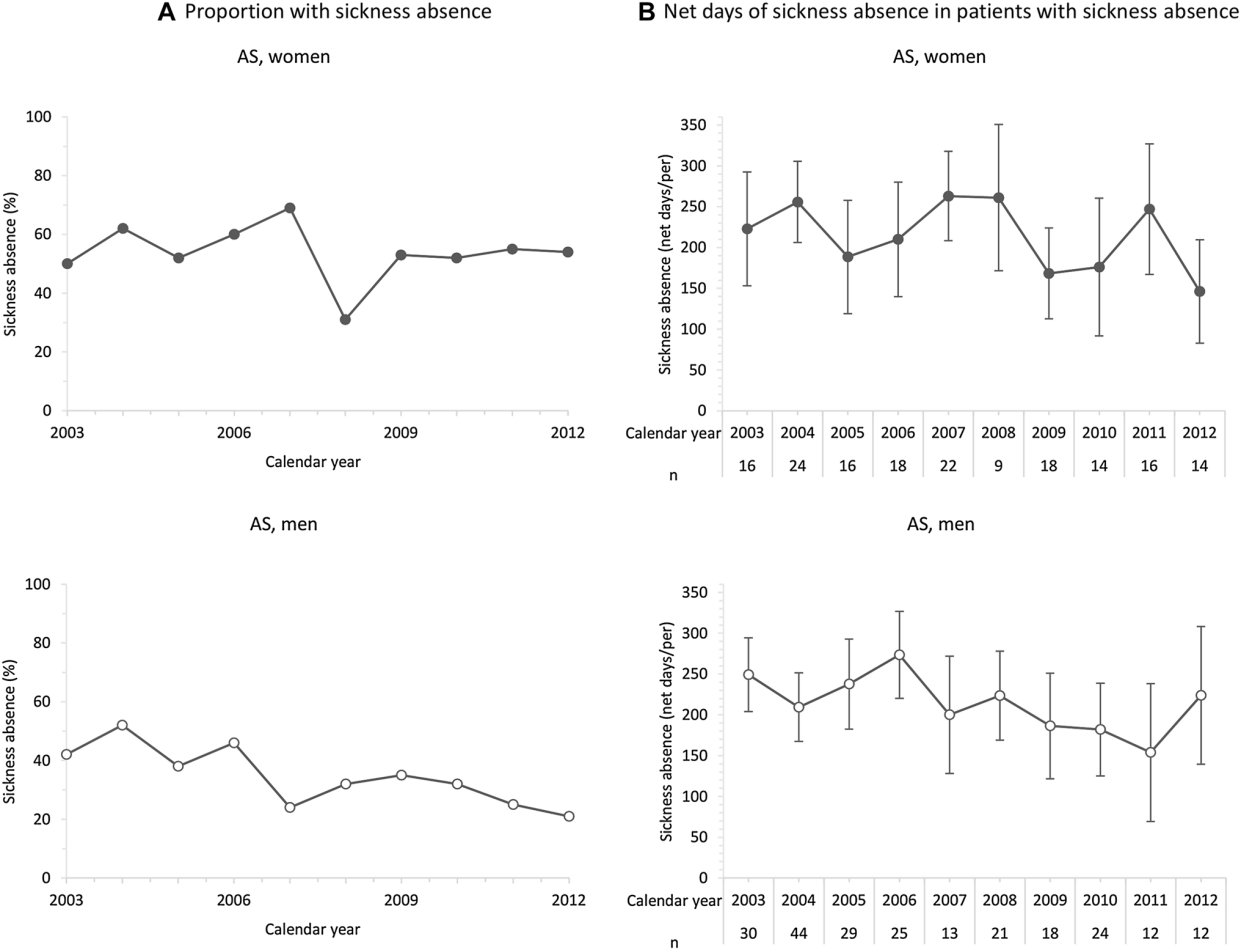
**a** Proportion of women and men with ankylosing spondylitis (AS), diagnosed between 2002 and 2011, with sickness absence during the calendar year following diagnosis. **b** Average number of net days of sickness absence for patients with some amount of sickness absence during the calendar year following diagnosis

**Fig. 3:**
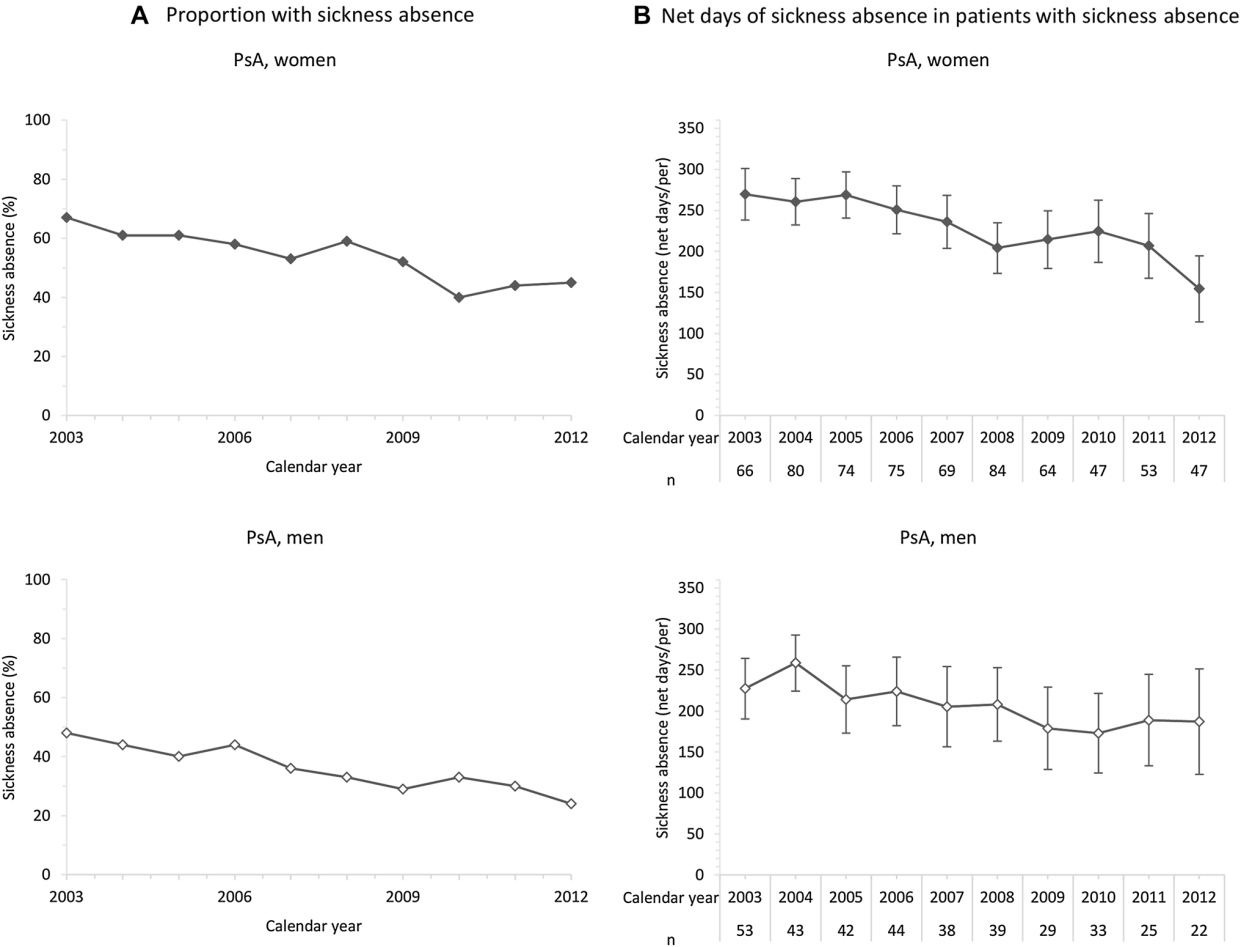
**a** Proportion of women and men with psoriatic arthritis (PsA), diagnosed between 2002 and 2011, with sickness absence during the calendar year following diagnosis. **b** Average number of net days of sickness absence for patients with some amount of sickness absence during the calendar year following diagnosis

When exploring if the change of net days with SA among the patients (Table 2) was related to proportion of patients with SA and or net days of SA among patients with SA, the proportion of patients experiencing some amount of SA during the calendar year following the index year did decline during the study period, except among women being diagnosed with AS (Figs. 2, 3; Table 3).

**Table 3:** Results of the linear regression models estimating the yearly change with respect to the proportion of patients with sickness absence (SA) during the calendar year following diagnosis and the average number of net days of SA during the calendar year following diagnosis in those patients that experienced some amount of SA during this period, in patients with ankylosing spondylitis (AS) and psoriatic arthritis (PsA)

	Proportion with SA (model 2)		Net days of SA in patients with SA (model 3)	
	β-Coefficient (95% CI)	*P* value	β-Coefficient (95% CI)	*p* value
AS, women	−0.4 (−3.1 to 2.2)	0.7128	−5.7 (−16.2 to 4.8)	0.25
AS, men	−2.7 (−3.9 to −1.5)	<0.001	−6.9 (−14.5 to 0.8)	0.07
PsA, women	−2.6 (-3.6 to −1.7)	<0.001	−10.8 (−14.9 to −6.7)	<0.001
PsA, men	−2.4 (−3.1 to −1.7)	<0.001	−7.2 (−11.0 to −3.4)	0.002

In PsA patients with some amount of SA during the calendar year following the index year, a reduction of the average number of net days of SA of more than a week per year occurred. There was a downward trend also among men diagnosed with AS, although it was not statistically significant.

## Discussion

The main finding of the study was that clear secular trends of declining SA were indeed present among men diagnosed with AS and among patients diagnosed with PsA. The secular trends in the reference cohorts were not at all as pronounced as in the patient groups. We were not able to identify a statistically significant secular trend in women diagnosed with AS. This may be explained by the lower number of patients and thus the lower statistical power in this stratum. The overall secular trend in men diagnosed with AS seemed to be primarily driven by a reduction of the proportion of patients experiencing some amount of SA during the calendar year following the index year. In PsA patients, the overall trend was related to reductions of both the proportion of patients experiencing SA during the calendar year following the index year and of the amount of SA, those patients experienced during the same time period.

Contextual changes in society with bearing on SA, general as well as specific for AS and PsA patients, have taken place between 2003 and 2012. Comprehensive changes concerning SA policies leading to a harsher SA climate were implemented [10, 11]. Indeed, slight declining trends were present also in the general population seeking health care, indicating that part of the reduction in SA among patients may well be related to changes with respect to SA policies. However, the secular trends in the general population seeking health care were substantially weaker than those observed for men diagnosed with AS and for women and men diagnosed with PsA, implying that changes with particular bearing for these patient groups could have occurred simultaneously. The results may suggest a shift in SA from longer, more secure periods towards shorter, patient-initiated and employer-paid periods. SA periods shorter than 14 days are not registered by the Swedish Social Insurance Agency and the findings of the present study need to be interpreted in light of this. In the context of the present study, it can only be speculated that beneficial disease-related factors may have played a role in the decline in SA over time. More efficient treatment strategies became available during the studied time window and this could have resulted in better patient functioning, in line with the findings of Maxwell et al. [12] and Lemos et al. [13] and as a consequence reduced SA as reported in the previous studies [14–17]. Other factors that could possibly be related to the declining secular trends in SA are shortened diagnostic delay, so that patients are diagnosed earlier in the progression of their disease, as suggested by Sørensen et al. [18]. An ongoing Swedish study performed in the same population does, however, dispute the notion of a decreased diagnostic delay in AS and PsA patients (Löfvendahl, 2017, personal communication). To sum up, multiple factors are likely to have had an impact on the amount of SA experienced by AS and PsA during the last decade. Whether the observed secular trends have been of benefit or harm to the patients cannot be answered with the current study design and further studies are, therefore, imperative to disentangle the underlying mechanisms and their impact on the patients.

An interesting sub-finding was that sex seemed to be more important for the experienced amount of SA than the actual diagnosis. During the calendar year following the index year, 54% of women, irrespective of diagnosis, experienced some amount of SA. The corresponding figures in men were 36% in AS and 37% in PsA. Furthermore, in 2012, the average number of net days of SA experienced by women diagnosed with AS and PsA during the calendar year following the index year was above 70, whereas the corresponding figures for men were below 50. This opens up for a discussion on how women and men that seek health care for similar diseases and symptoms are perceived by the health-care system. This is certainly a subject that deserves the attention of future studies.

The study had several strengths: it was population-based, relied on separate data sources for identification of cases and SA, and included a reference population to address whether or not general societal trends could explain the findings. Limitations inherent to register-based studies did, however, also apply to the present study. No information on the severity or activity of disease was available; neither did we know what treatment the patients received. Furthermore, no data regarding duration of symptoms were available. However, we focused primarily on newly diagnosed cases by applying a washout period, thus avoiding bias caused by carry-over effects from a more generous SA policy during earlier decades. Bias caused by differences between patients and referents with respect to their likelihood to seek health care was avoided by requiring referents to have had at least one visit to a clinic with a diagnosis set by a physician during the year. The results of the study can be generalized to working-aged populations in countries with comparable health care and social insurance systems as in Sweden.

## Conclusions

Secular trends of declining SA were present among men diagnosed with AS and patients diagnosed with PsA. Trends of the same magnitude were not observed among the general population seeking health care. Whether the observed secular trends are of benefit or harm to the patients is unclear and warrants further investigation.
